# Practicing Integrated Care Pathways in Norwegian Hospitals: Coordination through Industrialized Standardization, Value Chains, and Quality Management or an Organizational Equivalent to Improvised Jazz Standards

**DOI:** 10.3390/ijerph17249199

**Published:** 2020-12-09

**Authors:** Per Magnus Mæhle, Ingrid Kristine Small Hanto, Sigbjørn Smeland

**Affiliations:** 1 Institute of Health and Society, Faculty of Medicine, University of Oslo, 0314 Oslo, Norway; 2 Comprehensive Cancer Centre, Division of Cancer Medicine, Oslo University Hospital, 0450 Oslo, Norway; insmal@ous-hf.no (I.K.S.H.); ssm@ous-hf.no (S.S.); 3 Institute of Clinical Medicine, Faculty of Medicine, University of Oslo, 0318 Oslo, Norway

**Keywords:** integrated care pathways, cancer care, coordination, uncertainty, complexity standardization, improvisation, professional communities, professional networks, boundary spanners

## Abstract

The goal of coordinating pathways for cancer patients through their diagnostic and treatment journey is often approached by borrowing strategies from traditional industries, including standardization, process redesign, and variation reduction. However, the usefulness of these strategies is sometimes limited in the face of the complexity and uncertainty that characterize these processes over time and the situation at both patient and institutional levels. We found this to be the case when we did an in-depth qualitative study of coordination processes in patient pathways for three diagnoses in four Norwegian hospitals. What allows these hospitals to accomplish coordination is supplementing standardization with improvisation. This improvisation is embedded in four types of emerging semi-formal structures: collegial communities, networks, boundary spanners, and physical proximity. The hierarchical higher administrative levels appear to have a limited ability to manage and support coordination of these emerging structures when needed. We claim that this can be explained by viewing line management as representative of an economic–administrative institutional logic while these emerging structures represent a medical–professional logic that privileges proximity to the variation and complexity in the situations. The challenge is then to find a way for emergent and formal structures to coexist.

## 1. Introduction

Integrated cancer pathways (ICP) were introduced as a politically imposed reform in the Scandinavian countries during the period 2008 to 2016. The target was to achieve control on waiting times, improved quality of care, and increased patient satisfaction. The tools was describing a standardized treatment process for twenty six cancer diagnoses, defining normative times from received referral to start treatment and introducing new and mandatory coordinator positions and governance systems. Today, in the Scandinavian countries, cancer is the health field care where ICPs are most prevalent. The role of ICPs in cancer care springs from major developments in the field. Improved understanding of tumor biology, and advances in medical equipment, and information technology have led to more in-depth diagnostics and differentiated treatment, which in turn creates increased treatment demand and capacity [1,2,3]. These developments have been accompanied by an increase in expectations for integration and interaction, with relatively limited availability of resources and ability to cope with the fast dynamics of the development itself. Throughout this evolution, we see a tremendous increase in the complexity of structures, processes, and relevant knowledge, and thus increased specialization; meanwhile, more and more cancer patients are receiving or are candidates for multimodal therapy. Thus, a strong need for coordination emerges—coordination that includes continuous adaption and improvement [4,5]. Cancer care is arguably one of the most challenging medical areas in which to implement organizational principles oriented towards standardized chain-based processes. There is a lot of uncertainty, unpredictability, and complexity, both at the level of each patient and at an organizational level, not to mention the interdependence between the two levels. The problem addressed by our study is then to gain knowledge on by what kind of mechanisms the coordination through ICP is accomplished.

ICPs as a more general phenomenon have been one of the most pervasive phenomena in health care reforms during the last decades [6,7]. The phenomenon is known by several names [5] and specific content and implementation processes vary [8]. ICP is presented as a solution to meet the combined challenge of increased costs and quality demands in hospitals [9] and several studies have illuminated how ICP works [10,11]. Due to their obvious links to industrial management, ICPs have been accused of industrializing hospitals, shifting the emphasis away from professional discretion to cookbook medicine. The debate over the value of ICPs may seem like a struggle between the need for modernization and the defense of the values of medical professionalism [12].

ICPs incorporate three fundamental characteristics of industrial management [13]: standardization [14], value chain processes [7,15,16], and continuous improvement [17,18]. Standardization is seen in the increasing number of clinical guidelines, medical procedures, and diagnosis-specific action programs, all determined by evidence-based medicine’s best standard of care [19,20]. Combining guidelines, procedures, and programs into time-sequenced process descriptions, focusing on delivering satisfactory performance and outcomes for the patient and the hospital as an institution, is the equivalent of describing and designing value chains in an industrial analysis [21]. ICPs thus emerge as a kind of health care parallel to scientific management, or scientific–bureaucratic medicine [22]. Attached to the standardized processes are some key performance indicators expected to be the reference for accountability, adaption, and improvement of those processes. The link to industrially established processes such as lean and total quality management has been established [20,23]. ICPs are tied to a managerial philosophy characterized by the control exercised from the top of the hierarchy and executed through rational analysis, plans, and structural fit to achieve stability and reduce variation. The question is, however, whether this approach to coordination management can work in an increasingly complex organizational setting characterized by increasing uncertainty and a corresponding need for more coordination.

To address this question, we studied how coordination is performed and experienced through ICPs for three cancer diagnoses at four hospitals in Norway. We tried to grasp the naturally occurring processes, not just the formal ones [24], attempting to describe what people actually do [25] as a dynamic social practice [26] and identify how coordination happens regardless of organizational design [27].

In a welfare state model of health care, hospitals largely depend on accountability and rational planning encompassing standardization based on the economic–administrative logic. However, we wanted to explore whether there are also coordination processes that are dependent on other mechanisms than standardization and value chains and inspired by jazz we introduces the concept; the ability to improvise. This concept benefit from more spontaneous structures rooted in a floor level institutional logic. If so, we wanted to explore the interaction and dynamics between the processes and structures displaying standardization and improvisation, respectively. As mentioned previously, several of the elements included in our analyses have been addressed in recent literature about organization and health care: these include complexity, uncertainty, coordination, standardization, improvisation, organizational structure and design, and institutional logics. However, based on studying coordination practice in cancer pathway, we combine and connect previous insights from studies on these topics to deliver a unique understanding of process coordination in complex hospital structures under varying degrees of uncertainty and we identify through which types for organizational constructions it unfolds and are limited by. We are then contributing to a better understanding of management and organizational dynamics and encourage the development of more efficient ways of providing cancer care, and perhaps of managing other diagnoses as well, by applying the concept of ICPs. Thus, we deliver an insight that is crucial to managing pathway processes successfully and overcoming obstacles and challenges such coordination processes still face. Here, actually, lessons can be drawn from professional organizations outside health care and hospitals, although not from Taylorism in industrial organization.

### 1.1. The Need for Coordination—Division of Labor, Complexity, and Uncertainty

Division of labor is a core element of the capitalist mode of production [28,29], modern bureaucratic administration [30,31], and professional organizations [32]. The division of labor has a horizontal element with the labor process being split into several specialized operations. In both industry and bureaucracy, the competence and skills of each worker are specified independently and standardized through technology and rules. Such rules and technologies have thus been major tools for achieving the kind of coordination required within a company or a governmental administration. In addition to the horizontal division of labor, there is a vertical axis. The premises of technology, administrative rules, and plans, and agreements and even skills and deployment of discretion are removed from the immediate labor processes, establishing hierarchical levels that are controlling the content of the running coordination.

The classical coordination measures have, however, been tested by growing complexity and uncertainty. If coordination involves interactions to achieve specific goals, complexity implies an increase in the number of those interactions. The amount of interdependence that exists in a complex system may be more than the sum of the individual dependencies in that system, thus challenging each actor’s ability to cope with the total information necessary to act rationally. Dequech [33] connects this to Herbert Simon’s notion of actors with bounded rationality. He claims that the challenges of complexity may originate from either the real complexity in the system or context at hand or from the actors’ restricted ability to cope with this complexity. Accordingly, complexity does not merely stem from how complicated the operational interphase is or how complicated the compilation of necessary knowledge is. Complexity and the need for coordination may also emerge as a result of several interests or logics being present. In a case study of coordination in a hospital [34], conflicting interests played a core role in the analysis of coordination in formal and informal processes. However, in the field of organizational institutionalism, the presence of conflicting logics has been a focus of research and is important for describing the complexity in organizations [35]. Institutional logics refers to a set of cultural rules and cognitive structures that shape the premises for organizational behavior [36]. Actors filling specific positions may represent different logics. In this tradition, change processes in health care have been analyzed through the institutional logics of managerialism and professionalism [37,38,39,40].

In addition to the division of labor, the question of how to manage uncertainty has been a central area for research on organizational processes and structures [41,42,43]. Uncertainty can be described with degrees of uncertainty [33]. This could be understood as a continuum where uncertainty is characterized by a known probability of several possible outcomes or events happening as opposed to a situation with fundamental uncertainty or pervasive unpredictability with no known range of outcomes and no known probabilities [44]. Both Dequeq and Becker argue that situations of greater internal and external complexity combined with a higher degree of uncertainty will obviously pose a bigger challenge to traditional mechanisms of successful coordination.

The degree of uncertainty influences the degree of complexity because it affects the degree of predictability in the interaction between the involved actors and processes [45]. The modifying variable in this interaction is the degree of agreement between the actors involved. An agreement in this context is not a formal or juridical agreement but rather an institutionally established practice or a negotiated and mutual acknowledged way of perceiving or doing things at hand should be perceived or done, involving conflicting interests or logics. This is in line with Beckerts’s [46] assertion that institutionalization reduces uncertainty, or at least reduces the alternatives of thought and action when we are confronted with uncertainty. The presence of institutionalization in organizations thus facilitates coordination by reducing both current alternatives and the variation over time [47], thereby creating stability.

### 1.2. Coordination through Standardization, Organizational Fit, and Reduced Variation

Institutionalization refers to systems of thought and mechanisms of action externalized from specific human cognition and action. Systems describing standardized action, models of organizational design, and models for analyzing and improving quality are all tools to express institutionalized patterns in organizations. The building block in these organizational tools is the creation of a more or less universal classification [48]. Brunson and Jacobsson [49] emphasize the scientific foundations of modern standardization. They divide standards into two types: standards for what we do and standards for what we have. Similarly, Timmermans and Berg [50] distinguish between four subtypes of standards: design standards, terminological standards, performance standards, and procedural standards. Timmermans and Epstein [51] claim that the strong push for standardization in health comes from evidence-based medicine and the processes whereby professional organizations and regulatory bodies bring scientifically based evidence into practice guidelines, assessment tools, and standardized outcome measures. ICPs are seen as a standardization tool to implement evidence-based medicine [19]. Zuiderent-Jerak [52], however, argue that connecting standardization meaningfully to ICPs must be done in a hybrid fashion. He uses the term situated standardization, a hybridization inspired by city planning based on a specific analysis of what should be given space and what should be standardized.

Organizing can be seen as a way of integrating split and specialized tasks and functions, thereby achieving coordination [42,53]. The different organizing principles result from which dimensions to standardize [54]. Minztberg identifies three organizing principles of standardization: work processes, skills, and outputs. Deciding which to use is a question of finding the best fit between strategy, technology, product characteristics, and principal or market demands [53,55]. Galbraith [42] notes that there is a general historical development toward organizations designed to fit external requirements. One consequence of this is that structures have been redesigned along value chains [21], with resources bundled to accomplish optimal fit for costumers and users. Implicit in the choice of organizational design is a mode of hierarchical control [56] as it defines a structure for making decisions about the distribution of resources and for accountability related to the deployment of resources. Thus, as Miller and Power [57] note, the standardized system of accounting has to constitute an impact on organizational structuring.

The dominant model of hospital organization is based on the standardization of skills according to medical specialty and the establishment of a design based on the functional principle [58], what has been called a professional bureaucracy [59]. Though ICPs carry elements of organization based on function, product, and skill, they are dominated by an organizational principle of clustering tasks together according to a chain of events that delivers value for patients. The ICPs are constructed from classifications based on one or more diagnoses. The philosophy behind the ICP thus involves building standardized sequences of events, comprising standardized evidence-based medical procedures, supported by processes that continuously seek to reduce variation, thus delivering both optimal coordination and ability to cope with uncertainty and complexity. Coordination may be facilitated by continuous improvement and reduction of variation [26,60]. Variation, as a deviation from a standard, and improvement, as a mechanical process [24] aiming at reducing variation. One goal of ICPs is then to reduce variation and thus create increased predictability and stability.

### 1.3. The Processes of Coordination

The first obvious question when introducing processes like ICPs in an organization is: Should the coordination challenges be solved by creative redesign of the current formal organization to create a better fit? Several scholars [21,58,61] have discussed the search for re-designs contributing to a better fit. Numerous combination models that incorporate elements from different organizational principles have emerged including hybrids [62], matrixes [63], and front-back models [64]. However, some have questioned the narrative that has led to the search for such a redesign either as part of a theoretical discussion, an empirical case study, or a combination. Based on a case study of a radiology department, Symon [34] identified informal coordinating practices emerging on an operational level in the organization in parallel to formal procedures. Faraj and Xiao [65] analyzed the coordination processes in a hospital trauma department and described work processes that could be not be standardized in the way that administrative processes could. Klein et al. [66] conducted a case study of coordination in a trauma unit at a hospital. They found coordination practices that were a combination of some basic rules and procedures and some that they described as dynamic delegation in groups where membership is fleeting and tasks change often. Hoffer Gittell [20] studied how surgical teams doing joint replacement in acute-care hospitals coped with input uncertainty. She confirmed that relational adaptive processes played a major role. Rico et al. [67] discuss how team coordination affects team performance on a general level through what they call implicit coordination. This refers to how team members dynamically change, adjust, and adapt their contributions to attain common goals. Hendriks and Fruitier [68] discuss the possibility of aligning formal organizations with knowledge and point to the basic problem of finding a stable organizational model in fields where the knowledge base evolves quickly. Meier [69] studied coordination practices in three hospital units and found that all three were based on standardized documented work processes. When connecting the findings to the degree of unpredictability in the four cases, she concluded that in the unit with the highest degree of unpredictability, there was also comprehensive ad hoc coordination present. In a more theoretical contribution, Galbraith [64] developed the concept of lateral processes as an expression of cross-functional coordination based on weak direct instructions from the top down and more or less formalized cross-functional groups. Minzberg [54] introduced the concept of adhocracy which describes more or less spontaneous, multi-disciplinary, cross-functional, and informal structures accomplishing tasks or solving problems emerging from a specific situation or challenge. In the review presented by Martin et al. [70] the phenomenon of distributed leadership in networks is identified as decisive tool to accomplish adaptions and manage change. Lee and Edmundson [71] describe a general phenomenon of post-bureaucratic organizations where standardized structures are not predominant. These organizations are characterized by knowledge work and a desire to innovate and to align with opportunities emerging from the surroundings. They create space for networking, encourage team-based work, and have a flat organizational structure. They are evolving dynamically and resemble communities more than hierarchies.

This research all arrives at the following conclusion: In addition to coordination through standardization of structures and processes, complex organizations embedded in a context of uncertainty are characterized by the presence of informal adaptive activities that may directly coordinate tasks assigned by the formal line of management. However, as Stacey [72] makes clear, these systems are mainly self-organizing, non- or semi-hierarchical constructions with no formal borders to cross.

Organizational routines are recurring patterns of collective behavior, action, and interaction [44,73]. If there is a balance between predictability and flexibility to cope with uncertainty and complexity, organizational routines may be a more fruitful concept than standardization. Since organizational routines are embedded in the organizational structure and are practiced by actors with different roles and in different situations and contexts, there will always be an element of interpretation and customizing. These routines build organizational memory by creating a repertoire of past actions while at the same time remaining adaptable and customizable to fit new circumstances. It then gives legitimacy to a performance that may combine selective retention and necessary variation to cope with change. Routines may be expressed in standardized procedures. However, in practice, they are always developed and performed more or less independently from hierarchical governance and standardized systems.

### 1.4. Organizational Structures Facilitating Coordination

Routines have a coordinating capacity [44], facilitating social connections between people and groups [74] which in turn create shared understandings. However, most research on organizational routines has not been focused on the structural elements of connections that emerge from the routines. Feldman and Rafaeli [74] argue that social encounters are a relational aspect of routines and that they create ties among participants, producing networks. If organizational routines are useful for understanding how ICPs work as a coordinating instrument, we need to make some assumptions about what type of organizational constructs routines work through. Searching the literature, we identified four semi-formal structural elements.

The first is what is called collaborative communities [56,75] or occupational communities [76]. In the literature on organization, these can be traced back to Weber’s [30] concept of collegiality. Waters [77] elaborates on Weber, stating that in modern society collegiality is connected to the ideal of a society of equals specialized in different areas of expertise. Thus, decisions are made collectively in these communities, which exist independently but are still in some way related to the bureaucratic organization. Included in the concept of collaborative communities are communities of practice [78,79,80]. There are informal, emergent, and voluntary groups of professionals who self-organize to solve specific problems. These may be considered informal teams [24,67] and becoming visible through team meetings [20].

The second structuring element is social networks. Social networks in organizations are connections between individuals based on their position, relations, or shared events [81] or a set of actors connected by a set of ties [82]. Networks in organizations can facilitate transmission of tacit knowledge, simplify coordination, and prevent potential conflicts, and their function is problem-solving, knowledge sharing, or access-opening [83]. To survive, social networks within and between organizations have to be integrated into patterns of actions. They may have an emotional or instrumental function, and is often a mixture, representing personal and formal relations. Social networks are distinguished from groups and communities.

Integrators are the third structuring element. The concept of integrators was introduced by Galbraith [64] and it relates to a more or less formal position managing coordination across boundaries of a formal organization. Integrators are also referred to as brokers or boundary spanners [20,84,85] To perform their role, integrators must gain the trust from the groups and persons they are bridging. People in this role gain authority by facilitating cooperation between communities and successfully bridging boundaries. Their focus is operational, but they may also work strategically, depending on their impersonal role expectations or personal and entrepreneurial skills.

The fourth structuring element is physical proximity [86]. Physical proximity facilitates coordination by creating arenas for social ties, shared cognition, transfer of knowledge, and the emergence of common routines. Thus, physical proximity may foster low-cost coordination without involving the formal organization.

These four semi-formal structuring features are, at least as ideal types, clearly distinct from each other. However, in reality there is overlap in the spheres that they cover and the coordinating functions they perform. The overlap thus exists on both an ontological and an epistemological level. Networks build connections between communities of practice, bridging boundaries and structural gaps [87,88], and both communities and networks might include roles for boundary spanners and brokers [84,89]. Proximity may facilitate the emergence of communities and the relations that constitute networks [78,90,91]. Literature on organizational coordination discussing any one of these phenomena often refers to the others [75,79,82,83,84,92,93].

### 1.5. Improvising Coordination—A Supplementary Explanatory Approach

These alternative ways of structuring coordination will interplay with the formal structure. However, what all of these elements have in common is that their action and interaction can be standardized only to a limited degree. The processes are neither developed nor implemented top-down. The coordinating processes involve what we may see as the opposite of standardization, namely improvisation and experimentation. The concept of improvisation and its relation to organizational analysis is discussed in the literature, notably using the metaphor of jazz music [94,95]. A fundamental feature of improvisation is the absence of a time gap between planning and execution [96], as when something is created during a performance [97]. Improvisation is not the result of some existing specifications. During a jazz performance, it emerges through impulses and interactions between band members, instruments, and the audience. However, it also connects to the memory of how we used to perform, expressed through preexisting routines, and builds on ties to collective knowledge through networks, proximity, and collegial groups. Proximity facilitates the spontaneous element in joint coordinated action and building community around improvisation [95,98]. ICPs should be a processual learning device rather than a Taylorist device for standardization [52].

**Figure 1:**
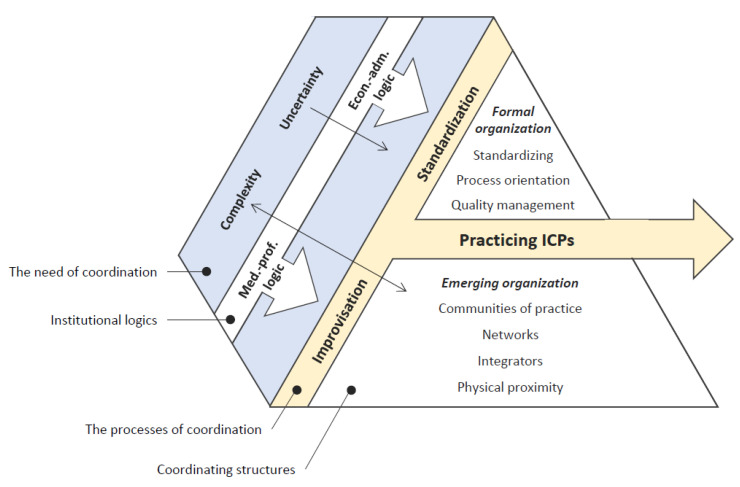
Relation and interaction between concepts.

The analytical and research-based concepts we have introduced to interpret the performance of coordination through ICP in hospitals are expressed in Figure 1. Uncertainty is framing relatively complex processes embedded in a mixture of several logics. Here the coordination accomplished in ICP unfolds in a combination of standardization and improvisation and through two types of structures—the formal and the emerging elements.

## 2. Materials and Methods

### 2.1. Description of the Field Studied

We anticipated that the patterns, the degree, and the features of complexity and uncertainty, and thus the need for coordination, will vary between hospitals and diagnoses. Accordingly, for this study we selected four hospitals, both community and university hospitals, and three diagnoses and studied all three diagnoses in all four hospitals to cover variations and common characteristics.

The selected hospitals are from two health regions and include the referral university hospital and a community hospital from each. Compared to the community hospitals, the university hospitals are several times larger measured in terms of patients treated, beds, and number of employees. In addition, their organization is more split into specialized units, their activity is spread over several locations, they have integrated research infrastructure and activity, and they have medical students. When it comes to cancer care, the scientific output is significantly higher at university hospitals, and in addition they perform radiotherapy, specialized centralized surgery, chemotherapy and diagnostics. At the community hospitals, activity is limited to one campus, the medical staff is mostly generalists, organization is less divided, and there are fewer levels from the bottom to the top. However, as university hospitals also serve as community hospitals, they have the same diagnostics and treatments as an integrated part of their activity.

The following variables were used to describe the complexity of and need for coordination in the three ICPs: patient volumes, degree of urgency, existing screening program, fraction of patients receiving multimodal therapy, and whether the surgical activity is separate from emergency activity. There are both general variations between the groups of patients and variations in the specific organization at the hospital level. An additional article will consider and explore the variation in coordination practices between diagnoses and hospitals, as well as the regional coordinating interaction. However, this paper concentrates on common findings regarding descriptions and explanations of the coordinating structures and processes of the ICPs identified across hospitals and cancer diagnoses.

### 2.2. The Data Sources

The object of investigation in this study is ICPs. The main sources of data were qualitative interviews and documents in the hospitals’ quality system. In each hospital, we had a contact person who gave us information about the hospital, procured relevant documents, and identified relevant persons to interview. We picked the informants based on these criteria: all key activities for all three pathways should be covered at all four hospitals. This means that we interviewed key medical personnel from outpatient units, surgery, oncology, pathology, and radiology department. Some of these were leaders; others had no formal management position. In addition, we interviewed patient coordinators, the majority of whom were nurses. We also interviewed some department leaders. Some leaders were responsible for more than one of the diagnoses and ICP; this was more seen in the local hospitals. Except for two interviews, all of sixty-six interviews were performed in the interviewee’s local environment. A relatively open interview guide was distributed to the interviewees ahead of the interview. Interviews lasted from half an hour to one and a half hours with a median duration of fifty minutes. All the interviews were recorded and transcribed. The distribution of informants is shown in Table 1:

### 2.3. The Research Process

**Figure 2:**
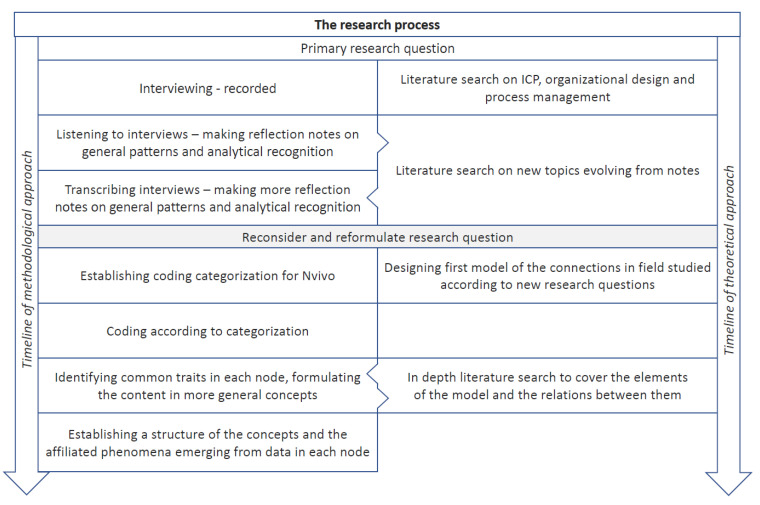
Schematic description of the abductive research process.

The initial focus of this study was the ICP phenomenon as a complex yet standardized set of procedures connected to the patients’ pathway through diagnostic and treatment episodes in and between hospitals. In the matrix structure made by the process-oriented ICP and the medical specialty-based line organization, we anticipated a tension between the process orientation and the formal organization. We wanted to describe how this tension was playing out and possibly being resolved. However, in a process that has been previously described in qualitative research [99,100], through careful listening to and analyzing the interviews we gradually had to reconsider what actually happened in the field and reinterpret what practicing ICP was about. We began to question the degree of standardization present and the role it played in accomplishing the coordination we observed. Parallel to this, we also questioned the role formal organizational structures played when dealing with horizontal coordination. At the same time, the coordination challenges between the vertical levels in the matrix were confirmed. However, the key challenge reported was not, as initially assumed, the lack of alignment between the formal organization structure and the horizontal ICP process. Based on this, we had to fundamentally reassess our initial research questions. We decided instead to describe the way ICPs unfold as an interplay between standardization and improvisation. To see how this dynamic played out in the real world, we identified several informal organizing elements and explored how they connected to formal hierarchical hospital structures. This abductive research process [101] is schematically described in Figure 2 and resembles what is labelled flexible pattern matching [102].

A source of knowledge of the field studied originates from the authors having long-term experiences of working with cancer care in university hospitals. This background influenced our general knowledge of field contexts, preparation and conduction of interviews and interpretation of data. Reflections on this were documented in a separate essay during the research process.

Analyzing our interviews involved multiple steps: writing notes about the recorded interviews, transcribing the interviews and making more notes, discussing our reflections with colleagues at the research institute and at the hospital as well as in a focus group of patient representatives. Parallel to these steps, we searched for relevant literature. In the previous chapter, we reviewed the concepts and contexts from existing literature that contributed to our analysis.

Our revised approach to the research matured gradually through these intertwined processes of analyzing our data and reviewing the literature. Consequently, when it was time to code the interview data in the NVivo system, we adjusted perspective, as mentioned previously. The revised perspectives for this study were operationalized through the step-wise creation of analytical nodes in NVivo. In line with Strauss and Corbin [103], the coding started as open coding and changed to a mixture of axial and selective coding. Each NVivo node was filled with rich citations illustrating the variable focused in each node. In the result chapter our ambition is to combine a presentation of vivid impression of empirical expressions of the analytical concepts with the result of a more synthesized presentation of the concepts. The first is accomplished through selected quotes and the second through a comprehensive set of points abstracted from the data material and presented in table form.

## 3. Results

The patient pathways in Norwegian hospitals emerged through a combination of national initiatives from professional associations and politicians and local efforts to design pathways, establish multidisciplinary meetings and employ patient coordinators. Evidence-based procedures and nationally standardized treatment guidelines for every cancer diagnosis are core elements of the officially approved cancer pathways. A monitoring system for waiting time was established. Cancer care coordinator positions were created and multidisciplinary team-meetings to decide on an individual treatment plan for each patient became mandatory. The ICPs represent a hybrid system containing elements from both the management and the professional level. The hospitals involved in the pathways were audited at the management level while the ICPs themselves were filled with content from a local professional level.

### 3.1. The Need for Coordination Work in the Face of Complexity and Uncertainty

To understand the coordination that takes place to provide an integrated cancer pathway we first identified the drivers lifting coordination on the agenda. The first driver is about complexity. Three of our informants expressed the development of increased complexity like this:

“During my time here, it is obvious that the complexity has skyrocketed. The examinations have become more extensive, even though we do not perform many more examinations in terms of numbers. But every single examination has become much more complicated, both on CT and MRI. And quite clearly there has been an increasingly greater pressure to respond faster.”

“The traditional part is based on either neoadjuvant treatment or treatment given in connection with having undergone surgical interventions, postoperative radiation therapy, hormonal treatment chemotherapy and so on. There is a pretty good, obvious path for each subgroup. It does get more complicated eventually; it is clear that we become more and more specialized. And further, if something happens to the patient, you have to obtain the images. And those images are often not described up against the images that are in our archive.”

“What was previously called breast cancer is now called ten to twelve different variants. So it is also within other organs. And the molecular biology has come into the picture and this with genetic changes. And mutations in the tumors and now for the last ten years the new cancer drugs have appeared. Those that are specific for tumors with this or that mutation.”

More generally our research material indicated that the complexity originated from three sources: medical conditions, logistics, and general hospital management, as seen in Table 2.

Not only the number and alternative kinds of interactions, but also the mutual interdependency and compatibility of information, expectations, and systems influence the total complexity. The fact that time and resources are limited also adds complexity. The presence of different but parallel structures and pathway alternatives connected to the same diagnoses also add to the increase in total complexity.

A second driver influencing coordination is uncertainty. Variations in key variables are predictable to different degrees, which affects the degree of uncertainty. Variations connected to patients are expressed like this:

"If one of my colleagues has read a referral and read a histological response and seen images and made a plan, and the patient comes to receive adjuvant treatment, then it may well be that everything changes when you see the patient and have talked to them. If they are old and frail and need half an hour in the office and actually have a lot of other diseases that didn’t appear in the referral and such, then you just have to change everything.”

“There are huge fluctuations, for example when we have very few rectum cancer patients referred here, and then we plan a lot of benign surgery for a period, and suddenly after a week a lot of cancer patients come in with short treatment deadlines. So it is a bit difficult to take the fluctuations into account.”

“There is variation, there are different urgency categories based on two conditions. The first is which condition it is, because we have peritoneal metastases from colon and rectal cancer. It is quite urgent. And then we have what is called pseudomyxoma which is a milder disease, which can have a fairly large spread in the abdomen, but is not as urgent because it changes very little over a few months, so it is reflected in how fast we give the patient an operation date. But we do give them a date and call them in for surgery provided that we are satisfied with all the available information, otherwise we must obtain more.”

As Table 3 summarizes, the variations are either medical-related, patient-related, or related to organizational conditions.

As for complexity, the aggregated unpredictability and uncertainty increases due to independent variations in several internal and external variables on both medical, patient, and system levels. The cumulative variation and uncertainty lead to increased unpredictability.

The overall complexity and uncertainty of cancer pathways creates a need for coordination, both for individual patients and for specific situations in the involved departments, which is connected to cooperation at the system level. For patients, this is experienced as delays and changes in treatment trajectory. Narrow timelines for standardized procedures and sequences with limited and partly unpredictable access to resources, are resulting in limited flexibility and opportunities for adaptation. The need for active coordination is reinforced by scarcity of human and equipment resources combined with challenges created by attempts to optimize allocation of knowledge and skills appropriately according to requirements of the patients. At the system level, the need for coordination depends on the system’s ability to manage the sum of the specific variations and complexities and make the necessary adjustments in each situation and case.

### 3.2. Coordination between Standardization and Improvisation

A glimpse from daily coordination is given from these three quotes:

“We work according to the principle that when we receive a referral, the patient will come in reasonably quickly. The patient coordinators reserve hours every single week and distribute them to the doctors and use those hours continuously. If we see that it gets cramped, we try to manage them outside the reserved hours or set up an extra outpatient clinic hour, and if there is fewer patients, then you can spend those hours on other patients. Otherwise we do not take fluctuations into account in a way. And we do have a backup, like when Easter is approaching and you cannot get an outpatient clinic hour, then a makeshift solution is to just admit the patient and start the treatment.”

“At least the respect for the logistics and what lies behind that kind of heavy decisions. Because you do not only connect the patient to a time, but plan in the direction of the patient receiving the right therapists, the right competence, you know, put together those teams, but the operation of the operation department are now… I have been given a capacity that I will fill, how that capacity is staffed beyond the surgeon, I have nothing to do with.”

“Yes, we get patients that either have a high risk disease or who have locally advanced breast cancer where we know the risk of having distant metastases is high. And we need to know that before we start the treatment, whether there is a spread already. Because then it is a completely different situation. Then we go from having a curative treatment to a life-prolonging treatment. And with those we know there is spread of the disease and then we need radiological examinations before we start life-prolonging treatment. And there we have major issues with waiting times to get examinations done. And we have major problems with waiting times to get responses after examinations.”

Based on our material a comprehensive picture of coordination activities in patient pathways might be described through: what is the mission, what is coordinated, how and by whom. We summarize this in Table 4.

The work of coordination can be summed up as compiling and transforming information between different sources through informal channels based on roles without a hierarchical relation to each other.

Explicit standardization is a coordinating mechanism. Documents and interviews also provided us with information about how standardization is experienced in ICP practice.

“A major change came through the adjustment of the classifications in 2014 when we received the latest WHO book. And this was due to the fact that there was a better molecular understanding of the tumors that we diagnose. So previously it was mainly microscope and some additional methodology that we call immunohistochemistry that looks at protein exposure. What has happened in recent years is that larger genomic studies have led to classifying tumors in a completely different way, and we are thus able to look into the subtypes of the diagnoses we are dealing with.”

“You standardize the treatment of rectal cancer related to how severe it is, so that if it’s a 3, 4B cancer then it should have radiation therapy, but if it is a T3 then you may manage with a simpler treatment and one week instead of five. And then there should be some time before surgery, and it should maybe be performed at [hospital] or it can be local, and if you have liver metastases then you should at some point operate that and have a little chemotherapy afterwards, and if you standardize such a path, then you can sort of build together a sequence where you first give chemotherapy for two months, then give radiation therapy and then you have to figure out what kind, and then since these are smaller than that, then you may want to operate the liver, and then you would like it to take eight weeks to that rectum surgery and then you give chemotherapy afterwards.”

From our material we deduced a more comprehensive picture of standardization connected to cancer pathways summarized in Table 5:

These characteristics provide a general picture: Patient pathways and the coordination process relate to standards. However, they are not treated as rules or absolute demands. They are more like a common framework or reference for practice. There is continuous negotiation, mutual adaptation, and consultation about interpretation. The standards are treated more like flexible, local routines adjusted according to individual patient needs, circumstances, and critique and based on local knowledge and alternative sources of authority. We were struck by the idea that this way of working had much in common with improvisation and thus looked for references to activities that could be classified as such. A few quotes from informants may illuminate this:

“It is often the case that you have to call, beg, ask, remind. Sometimes things go automatically, the physiotherapists come by themselves, but it is clear that in a system where someone thinks a little themselves and do what they want, and has other tasks in addition. I often believe that the surgeon wants to come, but then they get busy. They do have many tasks. So then you have to find other ways to go. Yes, and I think that as I gained a lot of experience, you will learn a little about how to handle different and how to handle the system, where the loopholes are. Where can I go?”

“That hybrid model is very difficult to handle. Because the acute tears down the entire planned structure that a cutting-edge expert need. Unpredictable—have to constantly jump around. All the plans you have made you have to plan again because they did not work. And these challenges we live with on a daily basis.”

“There are many similarities, but if you are thinking that there is a standard in the sense that you know everything about what kind of histological type it is based on, then you do not know. So there is always something to wonder about. Not least if there are patients with peritoneal carcinomatosis where the ovaries are not very prominent, then you will wonder whether it may be a primary ovarian cancer with spread to the peritoneum, or if it may be a primary peritoneal cancer, so it is not always a given that the ovaries are the starting point.”

A more general description of improvisation drawn from statements is summarized in Table 6:

To sum up, improvisational behavior from key actors along the care pathway makes it possible to handle cases, situations, and processes defined by scarcity, ambiguity, complexity, uncertainty, and unpredictability. Improvisation entails, among other things, adjusting the content of communication, form and timing, in tune with partners. Often some kind of improvised behavior is needed to arrive at the best common solution or conclusion. Improvisation thus also contributes to the development of mutual understanding and expectations which can inform future improvisations.

### 3.3. Structures Connected to Horizontal Coordinating Processes

We now turn to the organizing and structuring elements in which pathway coordination is embedded. At some points and for some procedures during the pathway, two or several organizational units are involved more or less simultaneously. At other times, there is an indirect connection. The outcome of one procedure or event can influence or be continued by another. Both situations depend on coordination. Coordination activities are performed—not perfect, not without ambiguities or tension. However, in lots of cases, situations and processes it does function. To phrase one of our informants:

“It is fantastic that it goes as well as it does, there are always fluctuations and I have to praise the people I work with, there is a great degree of flexibility because it is always, always full, in the outpatient clinic it is almost never free hours, so that they require a careful monitoring and control of all lists here.”

However, our informants reported that this coordination as taken care of to a small degree by the formal organization and by employees in formal management positions. Instead, this coordination is facilitated by four other structures that are present in the hospitals’ cancer care facilities: boundary spanners, networks, collegial groups, and physical proximity. These four structural elements are formalized and acknowledged in the performance of coordinating tasks to varying degrees. However, with all four of them, there is considerable room for content that employees involved consider to be appropriate.

First, we discuss the role of patient pathway coordinators, who serve as boundary spanners at and between hospitals. There might be several employees assigned to such a role, including physicians who might have a special medical coordinating role for patients or be assigned to a coordinating role at the institutional level for specific pathways. However, we concentrate on the formal patient pathway coordinators. There should be at least one connected to every cancer pathway at all hospitals. These quotes illustrate how they work, the first from a doctor the two latter from coordinators:

“If there is a need for an extra examination, then the most important thing is that the information reach the hospital and that we are able to process it quickly, and then there are the coordinators who are close to and who know the patient and whom to contact here at the hospital. And instead of trying to call around and try to get hold of a doctor on duty that may not have time to answer straight away, or at least we see the benefit of it going through the coordinator. Then it is mostly a person who responds quickly and knows the system in our hospital and knows where to go next.”

“I call a lot of patients. I can have, for example, patients who call me and ask “I had an emergency surgery because I had such stomach pain, and I wonder what it really shows, because I was supposed to get a check-up in the outpatient clinic.” And then I can see that this lady’s biopsy answer shows that it is colon cancer. And then I can do one thing, and that is to offer a quick appointment at the outpatient clinic for a conversation, but if we do not have an appointment before four or five days or six days, that is somehow the closest in time, and the patient will of course also know whether it is cancer or not. And then I try to get hold of a surgeon, preferably an operator to hear whether there is like a possibility, can you call the patient or can you see the patient outside the ordinary outpatient clinic time, because this patient would very much like to know.”

“It can sometimes really tangle, you have so many challenges and you kind of cannot untangle. And suddenly you kind of get like that, when everything loosens up it’s like it’s fun to be a coordinator. It is in a way when you get help and get answers and it resolves for the patient. Then there is almost a kind of euphoria (haha), it is like its own discipline. I think it is very liberating then, when things work out. You bang your head against the wall a little now and then.”

A more systematic description of this role may be drawn from our material as shown in Table 7.

Though the role of patient pathway coordinator is mandatory in cancer care, we get an impression of vast variation in how the role is performed and develops in practice. Some of those inhabiting this role in the pathways we studied relied greatly on improvisation in relation to both to cases and situations. They were certainly aware of existing standards but perceived themselves more as a kind of guide, moving patients along and keeping them informed, rather than as auditors and guarding a standard.

The second structure facilitating pathways was networks of professionals. Almost all of the informants talked about the extensive use of networks across the pathways, both within and between hospitals. These networks are clearly associated with certain kinds of formal meetings like multidisciplinary team meetings and regional pathway meetings. At the same time, they clearly exist independently of these formal networking events. Three of our informants expressed it like this:

“I have very consciously focused on creating or establishing this network. Because we are so dependent on cooperating here, so if I was constantly facing opposition in a way that someone would not cooperate or similar then it would have been terribly difficult to work together. So I am very glad to have the network. Also among the oncologists, I have become well acquainted with them over the years. We are the same people who meet every Tuesday. I also have a low threshold for calling them if there is something I do not. or wonder or. Or they call me, for example, and ask, ‘hey I do not quite understand this’ or ‘why did you do it like that’ or yes. So I feel that it is very helpful for me that someone know when I call. Or the other way around, they know they can call me if they would like to add another patient to the rectum meeting or something.”

“They are very easy to call to, the regional hospital. And we often have surgeons come to us for supervision when patients with surgical problems stay with us. So, we talk almost—not daily, but it is very easy to make another phone call. And the radiologists who present findings at the MDT-meetings are the same that describe our evaluation images during the palliative treatment, so we talk to them along the way. So we know each other well.”

“Yes, but I think I have to say that I know everyone who works in gynecological oncology in my region. So I know who I want to have contact with, but then I call and the person is not available that day and then you need to try getting hold of someone else then. We try to have collaboration meetings twice a year, and then we have national competence meetings.”

In Table 8 we summarize a broader picture of this phenomenon as it emerged through our interviews.

In addition to this, we got information about the dynamics amplifying coordinating networking. The role networking plays in formal meetings depends on the design of these meetings. Meetings characterized by one-way communication, like radiology presentations, do not contribute to networking in the way multi-disciplinary clinical decision meetings often do. A mutual enforcing effect is often reported between the latter type of meetings, whether formal or informal, and networks that exist independently of these meetings. Links are reinforced on an operational level as members learn more about each other’s knowledge, skills, and opinions. Over time, this contributes to trust, making it possible to build a joint holistic understanding of the pathway and its context. Stability of network membership over time and inclusion of members, not least from the fringes of the network, contribute to preserving and enlarging the network. However, the scope and strength of networks seems to vary depending on several of the factors mentioned.

The third structural element facilitating coordination that we registered was collegial groups or communities. While networks connect people across geographical and organizational distance, usually through more episodic contact, collegial groups emerge from more frequent and stable interactions, often characterized by proximity. They may manifest themselves through informal and semi-formal meetings illustrated like these quotes:

“In the gastro group we usually discuss within these internal groups before we recommend for a new method to be introduced. If there are input from clinicians that we consider not to be well enough founded in international guidelines and such, we are happy to discuss it with them after discussions within the group. So in the gastro group we handle issues internally in the group. In a flat structure, yes. We are also trying to make a plan for how we will do this.”

“The professional judgement is more peer to peer as we daily discuss casus and look up in literature. So if we consider something to be tough, others can have a look at the images. So I do experience a daily flow. That we communicate closely around the professional judgements. There are different groups here, and some are in one group and some in several. It works very well, and then you organize yourself within these. I do not know how others do it, but we have regular meetings and address what is urgent. It has probably been to get some pressure off from the leaders, so that we don’t have to go to the leaders all the time and say, ‘the gynecologists are not sending electronical referrals, can you fix it?’ Now it’s more like we are handling it by talking with the gynecologists, possibly via our leader. So it works well."

Findings related to collegial groups as an organizational framework for coordination in patient pathways are summarized in Table 9.

This kind of professional community association is distinct and not congruent with any formal organization. At the lowest level of hospital organization, however, there seems to be some overlap between formal organizational units and informal professional communities. On some occasions there is even overlap between local managerial roles and legitimate leadership of a collegial community.

The fourth structural element was physical proximity. This element, too, appears to have a clear and sometimes strong facilitating effect on coordination activities despite existing organizational borders in the hospitals. One informant expressed it like this:

“We receive images from other hospitals here as well, I think we have to talk about thing together. Discuss with our colleagues and also do quality checking. You must have proper environments and of course you can communicate from a distance, but often there is something about having a colleague, a neighbor and such, ‘Can you just have a brief look, what do you think about this?’ We also work closely with clinicians that come down and talk to us and ask us about things”.

We have summarized the content of this element expressed through our interviews in Table 10.

The effects of physical proximity were highlighted through contrasting descriptions of its opposite: distance. Distance was associated with anonymity, less predictability, poorer overview, and difficulty achieving necessary clarifications and professional reconciliations. However, it can be unrealistic to create proximity between all actual collaborators. Opinions varied concerning who was most important to have close (i.e., collaborating specialties versus members of same specialty, other patient coordinators versus other administrative personnel working with same patient group).

Multidisciplinary team (MDT) meetings are a particularly interesting phenomenon with a coordinating role. They are mandatory in cancer pathways as part of the process of clinical decision making and treatment planning. The meetings are, however, not visible in the formal organizational structure of the hospital, but have evolved according to the experiences of participants. They are an expression of the professional community at work, nourishing networks outside the meeting room and contributing to synergies with proximity in performing informal coordination as illustrated in a couple of quotes:

“We have our internal MDT meetings where the gastro surgeons participate too. We are very concerned with discussing the patients in a plenary setting and that no decision should be taken in the back room or between just two colleagues. We are obliged to do a joint review of the patients and reach a consensus and then stick to what we agreed upon. This process has its value. Earlier, each doctor determined his own strategy. However, later there has become an increase in both guidelines and joint discussions.”

“As a matter of fact, in my opinion, the professional level experienced in these MDT meetings has been high the whole time. What I have experienced as a clear advantage with the MDT meetings is that the colleagues learn to know each other better. We become buddies. So then there is no fear of calling your neurosurgeon and discuss a patient, or phone my urologist here at the hospital. Through these weekly MDT meetings we have really learned to know each other.

General findings regarding the coordinating role of MDT meetings are summarized in Table 11.

Our informants confirmed that MDT meetings are important events in cancer pathways. National recommendations and standards of medical judgment and alternative routes of process served as common points of reference. Overlapping and supplementary competencies are often present. Joint experiences, routines, and patterns from previous cases serve as a common memory. In addition, discussions about cases can include tangents, expressions of doubt, and critique. Decisions are ideally made through consensus in a spirit of joint responsibility. In several of the pathways covered by our study, the informants could not clearly identify who was in charge of these meetings. The specific way MDT meetings are managed varies and can change according to interpretations and negotiation of needs based on local experience.

### 3.4. Vertical Coordination and Its Limitations

One finding from our material is that both medical and logistical coordination were often achieved through interplay between these four mechanisms. Awareness and acceptance of this synergy require a combination of conscious and tacit knowledge among the involved actors. This includes professionals in managerial positions, especially on the lowest levels. However, topics related to cancer pathway coordination seldom seemed to be on the table at line management meetings. In the interviews, line management was seldom mentioned as playing a crucial role in performing coordination. In fact, in some cases, coordination efforts required approval or resources made available through decisions from higher managerial levels. The descriptions of these situations gave a general impression of a difficult and stagnant process, as illustrated as illustrated by a couple of our informants:

“You order those laboratory tests and set up an outpatient examination. I have regular meetings to plan the lists for outpatient clinic and surgeries. It is a complex matrix. This takes time to accomplish satisfactory. You have to use time, much more than I believed and it is much more time consuming than those guys on the top understand. They proclaim that we shall have predictability…. However, but you know we don’t know who the cancer patients will be from one week to another.”

“The one who has experienced a major problem returns to her department, and consider that there is not much to do about it. Any further efforts then depend very much on the engagement of the pathologist experiencing the problem. More rapid processing of these biopsies from mamma-cancer could be an option. However, that would depend on access to more resources. So then everyone could turn to his or her leader and explain the problem, and there it will rest in peace, so definitely it is a need for someone on a high level to be more aware of the problem.”

This phenomenon is more systematically elaborated in Table 12.

These descriptions suggest that the involvement and support of line managers in solving coordination challenges at the institutional level are dependent on the interaction between the informal systems of professional communities and networks and the formal line management and leader meetings. The vast majority of respondents experienced that it was much easier to get support for adaptions, improvements, and innovations from their peers than from their superiors. More, the agendas of these formal meetings appeared to consist of issues decided from above related to finance, Human Resources (HR), or administrative governance topics. The necessity for attention to coordination needs does not seem to be pervasive or dependent on consultation with even higher levels which either lack the managerial capacity to deal with such issues or do not prioritize them.

## 4. Discussion

We have shown that the degree of complexity and uncertainty characterizing cancer patient pathways is high and tend to get higher. Based on interviews with key clinical personnel from four different hospitals in Norway we have identified the practices and actors involved in coordination. Our main conclusion is that coordination through industrialized standardization, value chains, and quality management is not sufficient due to non-controlled variations and individual adjustments for the patients. Coordination on a daily and individual case basis requires a culture of, and a skill for, improvisation.

Lillrank et al. [61] approach the challenge of managing and coordinating health care operations by dividing processes according to degrees of urgency and specialization. They describe seven categories and propose that the solution is to align processes with similar types of demand and add operating logic. Cancer pathways, however, contain activity in all seven categories and in addition, in the case of most pathways, are integrated into hospital organizations competing for the same resources. When several interrelated processes coexist and are based on different logics it is not possible to optimize them all simultaneously [104]. Avoiding complexity and, to some extent, unpredictable variations is therefore not only impossible but also an inappropriate perspective. The real world is not standardized [51] and variations may be necessary, not least if the patient is to receive the best possible personalized therapy according to her/his needs and wishes and adapted to the situation [47], but also simply to optimize treatment quality under uncertainty [105]. Accordingly, there will not be a perfect fit in formal organization establishing formal lines and fora of command and coordination to efficiently serve the purpose of coordination for all interests [72]. Despite variations in the specific organizational models of the hospitals we studied, we could not recognize any alternative organizational model that played a decisive role in accomplishing pathway coordination. Our analysis in this study acknowledges that the standardized descriptions of pathways and formal organizations play a role as a context. However, on a daily basis, for cases and pathways in need of active coordination, these structures do not play a major facilitating role. Rather, we have identified four semiformal structural elements that play a decisive role in performing necessary coordination: professional communities, social networks, boundary spanners, and physical proximity.

The four elements identified in pathway coordination have all been described in the current literature, both theoretically and empirically, but never in a comprehensive setting. What we have added is the importance of the four factors and how they act separately and in combination to facilitate coordination in processes characterized by complexity and uncertainty. However, we find spare evidence of coordination facilitated by the four structural elements being discussed or represented at the level of the hospital management team. We argue that the explanation is twofold: First, though the four elements may have a formal or physical expression, the way they perform coordination is not incorporated, and therefore hardly visible, in the formal organization or the management team. Secondly, the formal organization of a hospital and the processes containing the managerial arenas mainly represent the economic administrative logic. This logic is expressed by the accounting and auditing systems [106] while patient pathways mainly represent the medical and patient-related logics. These two explanations for the lack of contact between the formal and semi-formal structural systems are probably connected to and reinforced by each other. That means that if medical and patient-related logics are incorporated in a management system, it happens through a process of co-option whereby the medical professional logic tends to stay at a rhetoric level [22,40]. A revised structure and process believed to create a better fit between organizational structure, process, strategy and outcome do not solve this tension and lack of connection between the two systems of structures.

The processes of pathway coordination carried out by our four semiformal, emerging structural elements may be dependent on adjustments and access to resources or contextual regulations, or infrastructure governed by the formal organization. On such occasions, the coordinating mechanisms of the informal system often fall short. The processes in the emerging structures strive to achieve the attention and trigger the necessary action. The lack of acknowledgment of the self-organizing system is highlighted by den Herder-van der Eerden et al. [107] in a study of integrated palliative care and in a study by Pine and Mazmanian [108] on the implementation of an electronic health record. Therefore, some kind of integration and coexistence of the two structural systems is needed. Martin et al. [70] notes that they are mutual dependent to succeed. This integration would also deliver a legitimate demand for accountability for the outcome of the coordinating processes to higher administrative management levels, filling the expectations from the principals to their agents [109,110]. The contribution to establish a fruitful coexistence lies in improved understanding of the interplay between standardization and improvisation in hospital organizations.

Scholars of organizations including both perspectives report the presence and a role of standard elements in addition to memories, references, and routines. Practicing improvisation is then about developing skills to see the opportunities and develop connections that emerge through the pathway processes and for the involved actor to be trained to listen, interpret, and build on to the contributions of others while keeping in mind the recognizable standard elements. This is what Austin [111] calls transactive group memory, which is aligned with our description of the dynamics of coordinating MDT meetings and confirms the analysis of Oborn and Dawson [88] in their study of cancer MDT meetings. In this way, formal patterns and the emerging opportunities merge, coordination is achieved, and the patient is satisfied with their pathway experience.

In accordance with our findings, several scholars highlight the necessary interplay and coexistence of both the formal and the emerging structures of work processes. Banks et al. [24] describe how top-down coordination must be actively supported by bottom-up processes. Hoffer Gittell [20] notes that the presence of established routines supports the work of coordinating meetings and the work of boundary spanners in the presence of high uncertainty. Meier [69] describes how the relation between planned coordination and practice is performed through improvised processes of moving things around and letting things happen while den Herder-van der Eerden et al. [107] point to the interplay of nourishing the professional core teams and informal network parallel to necessary support from external authority and standardized pathways. Finally, Pine and Mazmanian [108] revealed the danger of underestimating the importance of the informal artful coordination of clinicians in the process of implementing electronic health records.

However, it is tempting to try to look for a transcending model implying structures that integrate the coordinating capacities of boundary spanners, networks, and communities into the formal organizations and the managerial system. Scholars [75,92] argue that the solution is to actively manage the coordinators, networks, and communities of practice, incorporating them into ordinary management processes [112]. Improvement of formal design is also the answer delivered by a study on coordination of cancer care [113]. Our analysis does not lead to the same conclusions. Semi-formal structures will only survive and flourish when they are allowed to exist relatively free from formal organizations and management. The study of introducing cancer-genetic pathways in England [70] as well as a recent study from German military supports this view [114]. This reasoning connects to the presence of separate, distinct institutional logics and tries to avoid blurring and coopting mechanisms between them. One of Stacey’s [72], core arguments, based on his work on complexity science and organizational dynamics, is about the need to keep self-organizing local emerging types of organizational processes separate from formal hierarchical command and control. The organizational space created by this separation corresponds to the organizational slack that according to Clegg et al. [115] is necessary to achieve organizational learning.

There is a third argument for not incorporating the coordination processes attached to the four emerging structural elements into the management system. These emerging structures rely more on improvisations and less on standardization and thus represent a more organic and less mechanical approach to coordination than formal organizational structures. The structuring mechanisms of professional communities and networks build on different types of leadership. In improvisation, there are hardly any formal leadership positions or, if they do, the positions change over time and in relations to circumstances or are difficult to recognize [116]. It is a distributed leadership characterized by being voluntary, informal and organic [70]. In the formal hospital organization, on the other hand, there is a clear rigid hierarchy.

Lateral processes [64] and distributed leadership [70] should be encouraged, and adhocracy [117] should have a place as a core element of coordinating pathways. At the same time, these forms of organization will be embedded in the formal organizational structure based on function and work chain. Our study as well as the study of Martin et al. [70] also based on cancer pathway cases-studies, indicate that allowing the two types of structures to coexist and flourish on their own premises, but at the same time interact, is a difficult balance to achieve. It is a question of accepting the presence of locally based self-organizing processes [72] and practicing self-managing processes of post-bureaucratic structures [71] inside a more traditional machine-bureaucratic framework. In the end, this may be the only way to succeed in coordinating care pathways in hospitals facing increased uncertainty and complexity. The conversation through which this interaction occurs [118] may combine two way scholars have proposed to deal with pervasive uncertainty: formal and informal institutionalization [46] and creativity and so-called animal spirits [33].

## 5. Conclusions

We have shown how ICPs in cancer care for three diagnoses at four Norwegian hospitals were practiced through a balance of standardized and improvisational means. We have addressed how these can be managed to create connected processes and integrate relevant knowledge to meet the challenge of inevitable uncertainty and complexity. There is room for improvement in terms of how standardization and process-oriented structures are designed and applied. The same is true concerning improvisational skills. However, we claim that the presence of both improvisation and standardization is not the main challenge connected to needs for coordination caused by complexity and uncertainty. The main challenge is to accept and understand the two different ways of structuring with their unique premises and to acknowledge and respect the crucial role of emerging self-organizing coordination processes that we have identified through our analysis.

One conclusion from this study seems to be that the standardization, value chain organization, and Total Quality Management (TQM) inspired by industrial management development do not provide satisfying explanation of how the hospitals we studied performed coordination of integrated pathway processes in cancer care. However, we observed that both those opposing industrial sources of learning and those promoting them seemed to lean on these tools of standardization, value chain redesign, and TQM. The emerging structures and improvisational processes we found that were actually decisive in explaining the practice of ICP can also be found in industrial contexts [71]. Therefore, it is not a matter of industry and health care being two different worlds and that there is just limited learning between the two. Experience and knowledge might fruitfully be exchanged between industry and cancer care.

The design of the current study lends itself to more research. We have focused on identifying processes on the meso-level using material from one type of process occurring in hospitals; it will obviously be valuable to supplement this work with more detailed analyses covering more elements in these processes, as well as other types of processes in hospitals. However, more important than research in this field is experience and practice that actively stimulate the dynamics between actors involved in the pathway, which is the crux of our study. Although improvisation, by nature, is nearly impossible to control and manage in a traditional way, it should nevertheless be acknowledged, made visible, facilitated, and encouraged. The most valuable research will then probably be research that looks at experiences with improvisation and attempts to learn more about its fundamental ambiguity. The studies of Martin et al. [70], Oborn and Dawson [88], Zuiderent-Jerak [52], den Herder-van der Eerden et al. [107] and not least of Schulte et al. [114] are a good example of this.

In conclusion, lack of coordination in health care is not caused mainly by failure of a formally governed and strictly managed system. The problem is that these systems have to be practiced in a way that simultaneously nourishes and recognizes the emerging social structural relations and more informal improvisational processes that we have identified. Achieving this kind of coordination will be at the core of the art of managing complex processes as ICPs and, at the next level, complex hospitals. Awareness of the coexistence of formal hierarchies and emerging social interactions is lacking, and more models and practical examples should be encouraged. This dance of coexistence between two models of organizational practice can be summed up in this refrain from an old jazz standard: “It don’t mean a thing if it ain’t got that swing.”

## Figures and Tables

**Table 1:** Number of informants from the participating diagnoses and hospitals.

	Hospital 1	Hospital 2	Hospital 3	Hospital 4	Total
Ovary cancer	5	4	2	1	12
Breast cancer	6	4	2	1	13
Colorectal cancer	10	9	4	3	26
Two or three cancers	3	1	4	7	15
Total	24	18	12	12	66

**Table 2:** Which conditions create complexity with regards to the coordination of the patient pathways?

Medical Conditions	Logistical Conditions	Hospital Management Conditions
Novel sources of diagnostic information and increased in diagnostic sub-classification	Non-compatibility and non-alignment of clinical and administrative data systems and information sources	Diagnoses/conditions include both acute and elective activities and with different patient volumes that all claim priority
Increased interdependency between diagnostic tests and treatment methods	Composite requirements and expectations for communication and involvement of patients and professional partners	Interaction between hospitals with different degrees of specialization and organizational models
More patients candidates for multimodal treatment (i.e., combinations of surgery, radiotherapy, and medical oncology)	Non-congruent principles for organizing and actors in pathway meetings	
Greater interdependence between a wider set of more specialized competences		

**Table 3:** More or less predictable variations creating uncertainties affecting patient pathways.

Variation in Medical-Related Conditions	Variation in Patient-Related Conditions	Variation in Organizational-Related Conditions
Assessment of criteria for referral and prioritization	Patients’ life situations defining needs and prerequisites for types of treatment and rehabilitation	The roles and areas of practice that the involved professionals and specialists cover
Patients’ general medical condition (molecular classification, cancer stage, spread/metastases, aggression, co-morbidity)	Patients’ need for information and involvement	Access to infrastructure and shared clinical resources
Degree of medical emergency	Variations in the number of patients referred	Mutual understanding of need for information, competence and procedures
Risk of complications	Patients’ choice of hospitals	Administrative urgency due to unsatisfactory monitoring data
Need for supplementary information to make medical assessments	Patients’ experienced urgency	Access to information from other parts of the pathway
The sequence of procedures prescribed		Prioritization criteria available

**Table 4:** Coordination activities in patient pathways.

What is the objective of coordination?	Achieve coherence in efforts/resources to cover the same activity Align a number of activities (examination, interpretation of diagnostic information, treatment) Collect all relevant knowledge about the patient before decision making Provide each patient with specific compiled information according to her/his needs Harmonize expectations and knowledge on opportunities between actors and interests
What is coordinated?	Knowledge (formal and informal) about the state of the organization – institution and external collaborators Knowledge (formal and informal) that may influence the pathway for individual patients Bridging organizational knowledge and patient knowledge Bridging medical premises and professional decisions and logistical needs and context Internally oriented: check-out and mutual adaptation between different contributors Externally oriented: adaptation to variation and unpredictability from the environment

**Table 5:** Standardization in patient pathways.

What is the objective of standardization?	A way to create a common language and framework for coordination by common agreeing on requirements and expectations on content, time and relation The standard provides legitimacy to specific actions and patterns of action The standard creates a framework for building trust (but also a basis for mistrust because different ways of interpreting standards may raise questions about the behavior of “others”). The process of developing a standard is therefore important for building trust
What is standardized?	Procedures (how to carry out specific actions) Processes (sequence of procedures, waiting time, organization, decision premises) Classification of disease (inclusion criteria, criteria for stage, grade and prognosis of the tumor) Treatment of disease
How is standardization expressed?	National documents with approval from central authority Institutional documents Clinical trial protocol No written routines related to processes IT applications like key target measures, flow charts, guidelines, checklists, templates
Conditions that lead to variation in the use of standards	Caused by local adaptations How detailed the input of patient information and patient features are in relation to what the standard requires Local clarifications, interpretations, and adjustments of how national standards are practiced The degree of knowledge, skills, and ability necessary to enact discretion and the role responsible for this (e.g., translator, leader, facilitator, controller) Individual patient cases challenging standards if they are considered outliers, or if there are doubt of quality and representativeness of tests(e.g., tumor location, metastasis present, and molecular characteristics) Caused by acceptance of general standards Disagreement about the evidence for some standards Ranking and highlighting standards to express that other variables (possibly also with standards) should be subordinated or adjusted to achieve the overarching standard. Possibility of by-passing some standards Mismatch tension between national standards and local practice: a) formal standard does not fit local circumstances or b) standard is implemented in one part/phase, but not other parts Failure to update standards on regular basis or local delays in adaption on new standards

**Table 6:** Improvisation in relation to coordination of patient pathways.

What are the objectives of improvisation?	Find solutions to peaks and bottlenecks in real-time or proactively, in relation to something that needs to be done to be prepared to a situation (individually or institutionally) Bridge gaps in pathway processes that may require particular arrangements (due to dissimilar systems, relative capacities, competences and organization) To build professional consensus, which requires interplay of knowledge between different specialties/professions Adjust logistics and professional choices to particular conditions tied to individual patients (which are covered to lesser extent by standard repertoire) Adjust procedures to cases that are not representative Improvisation as a learning process for development further cleverness to improvise, developing perception of the type of particular process/case, and establishing updated experience based routines
How is improvisation expressed?	Communicated through: ○ Electronic channels, including email ○ Casual or informal meetings, conversations, etc. ○ Formal meetings, such as multidisciplinary team meetings Counselling, facilitation and negotiation and leadership role The entrepreneurial role of some of the involved parties, requiring a combination of maneuvering with overview and detailed knowledge (of variations, loopholes, flexibility, actors, and network) and skills (independence, perseverance, knowledge of common “language,” freedom to move, ability to handle complex information)

**Table 7:** Coordination activities by patient pathway coordinators.

What are the manifest objectives of coordination?	Raise awareness of the need for coordinating access to resources and information, and thus logistics, thereby release both doctors and mercantile staff from doing unnecessary work Fill the role of a nexus—both within the hospital and between hospitals Create predictability for the patient Connect events in processes, and establish expectations for fulfillment
What are the latent objectives of coordination?	Bring non-formalized information about individual patients into decision-making processes Establish a possible vantage point for system learning and contribute to improvement
Development of the coordinator role	Great possibility for role adjustment based on situation and context in pathways and the coordinator’s prerequisites Often performed in cooperation with individual clinicians who perceive the role as an opportunity for both influence and relieve Some have accumulated data, such as self-developed overviews etc., which ease coordination Over time, there has been maturation in the deployment of the role in many units and pathways

**Table 8:** Networks as an organizational framework for coordination of pathways.

What is the manifest objective of networks?	Serve as a framework for obtaining supplemental information or verifying interpretations of information Provide access to capacity, time and resources of others Communicate adjusted priorities, progress, and needs Serve as a basis for mutual reconciliation of needs, requirements, and expectations Make handling of complex relationships manageable Provide a channel for accessing areas of knowledge that are only needed in special cases (e.g., anesthesia/pain, internal medicine, physiotherapy, etc.)
What is the latent objective of networks?	Serve as a framework for dynamic learning Serve as a framework for inter-disciplinary relations Compensate for limitations of more formal meetings Serve as a framework for social knowledge, recognition, and consideration for each other Compensate for lack of information on particular patient needs or conditions
How are networks expressed?	Form: Informal in-person chat Meetings which are initiated for other purposes Phone call Ad hoc organized interdisciplinary meetings addressing specific issues, professionally or logistically Notes enclosed in electronic documentation/communication Content in network contact: Professional assistance, interpretation, or problem solving Information and assessment of specific patient cases Logistics information and clarification Assessment of systemic and institutional contexts
Conditions that may affect formation of networks	Physical proximity in the work situation Size of the organization Actors’ previous careers Actors’ roles and/or personalities Interaction over time Proximity to profession Participation in regular and more formal meetings (e.g., MDT, pathway manager meetings, coordinator meetings, admission meetings, operation plan meetings, visits, etc.) Courage and ability to act by crossing formal structural boundaries Interest in spending time on professional-social informal processes

**Table 9:** Collegial groups as an organizational framework for coordination in patient pathways.

How do collegial groups arise, how are they constituted, and how do they reproduce?	Groups are constituted around simultaneous work on common tasks and problems or similar issues or direct interaction in the execution of procedures; they work as a unit Clarification often takes place in processes characterized by consensus, and in this community solutions to problems are sought horizontally before being lifted vertically Physical proximity is important for creating rich meetings that encourage and maintain collegiality Social relations develop from professional relations creating an infrastructure for further growth Shared knowledge and language (cognitive proximity) help to reproduce the group and stimulate effectiveness. This language must be expressed verbally, not just in writing, to allow for interpretation, adjustment, and improvisation. This language also provides implicit access to a holistic understanding
How are collegial groups expressed	Can include professionals from similar or different fields, specialties, and roles, but in a format where they receive equal recognition despite differences (as opposed to pre-visit meetings or radiology meetings, for example) No formal leadership and often also hard to identify informal leaders Exist both within and between organizational units and sometimes between institutions
Conditions that may affect collegial groups	Formal meetings (such as MDT, complication meetings, common bed units, operations, shift teams) can give rise to spin off groups and to some extent presuppose such informal communities in preparations and follow-up. Collegial groups require such communities, which are mostly based on professional issues but sometimes overlap with logistical networks Semi-formal and self-organized meetings, including meetings to discuss interpretations of preparation or share results from fresh research, interdisciplinary Friday meetings to follow up cases over time, can help build collegial relationships Collegiality often spreads through informal contact, without meetings or formal referrals (and without the contact necessarily being documented in journals etc.) A relatively flat structure provides a framework for interpreting more standardized guidelines such as action programs The context for community in a collegial group can be joint clinical decisions, joint research, shared quality registers, or collaborative pathway processes

**Table 10:** Physical proximity as a framework for coordination in patient pathways.

What is the manifest objective of physical proximity?	Expressed objectives: ○ Strengthen the interaction between collaborating specialties ○ Strengthen the coordination between coordinators in the same pathway Develop interaction in relation to individual patients
What is the latent objective of physical proximity?	Provides a low threshold for informal exchange of information between partners who must coordinate their efforts in situations, cases, or over time—professionally and logistically and in collaboration on clinical studies Allows for common informal and semi-formal physical meeting places, which also constitute a free space on the border between formal structures Helps establish social relations and mutual respect Creates room for mutual learning Permits more meaningful interaction between people than what is registered within formal organizational settings
What creates physical proximity?	Closeness between offices, shared water coolers, meeting places in common patient areas or patient processes Smaller overall organization and building size are contexts affecting proximity Establishment of proximity partly as random and partly as conscious historical processes Some historically close proximities continue to function—at least after separation for a while

**Table 11:** Coordination in patient pathways via multi-disciplinary team (MDT) meetings.

What are the manifest objectives of coordination?	Compiling knowledge and information that together provide a basis for clinical decisions Creating a meeting point for coordination of decision information that has an effect in relation to quality and efficiency in content and in logistics Increasing trust in decisions due to the shared decision making involving all relevant professional colleagues
What are the latent objectives of coordination?	Encouraging increased precision in referring and agreement on mutual expectations to content of decision basis Building transverse common understandings and mutual expectations over time (applicable to both clinicians’ requirements and diagnosticians’ expectations and, conversely, diagnosticians’ understandings of how their own feedback may influence treatment)

**Table 12:** Initiative from the bottom up—elevating problems in the line.

Descriptions of what happens when attempting to elevate problems	New opportunities created by new technology and knowledge are not necessarily discussed upwards but may require some resource allocation Medical considerations, prioritizations, and dilemmas that require transverse assessments, while not topics that are elevated, may claim resource allocation Bottlenecks in operation and investment are often elevated year after year—persistence may lead to success Issues might reach the immediate leader level, where there is also a connection to the medical, while attempts at further elevation stall
Interpretation of these challenges	The system is cumbersome to maneuver upwards in. The size of the organization may influence these challenges. Physical proximity to line management also plays a part There is not always a clear understanding at ground level how processes work further up the administrative line The line is not perceived to be a forum for transverse coordination, but rather for direct administrative issues; when transverse coordination requires resource adjustments in one unit, the authority and responsibility lies with the line management; however, do not have the authority or managerial capacity to accomplish the transvers coordination Individuals in the management-line are unfamiliar and unconfident in taking coordinating roles—both professionally and procedurally Changes that are implemented after elevating problems are perceived as symbolic Elevating problems to management level requires a greater formalization than is found in collegial groups and networks and forwarding claims are therefore inhibited External medical lines connected to administrative lines in cases that affect coordination ability and bottlenecks, lingers out The line may be a part of historically inherited, established structures that are characterized by power struggles and territory markings, and thus not suitable for solving coordination The role of certain coordinating actors in elevating cases tied to coordination needs may be unclear
How is lack of success in supporting coordinating needs managed?	By obtaining consensus and support in the collegial community (e.g., transverse checklist for MDT meeting preparations) and then elevating the issue to line managers rather than addressing the topic in formal meetings Breakthroughs are sometimes experienced as being dependent on the background and random interests of the managers up the line, as well as their network relationship to the ones raising the case (possibly external stakeholders/clients who have put it on the agenda) Coordination through formal management structures a may seem most effective when the units needing coordination are close organizationally and not far apart Significance of separated or integrated hierarchies on medical and administrative issues Line managers often coordinate via their more informal role in medical collegial networks, which may support the line manager role
